# Epidural hematoma, a positive or negative prognostic factor? Letter to the Editor in response to Khaki et al.

**DOI:** 10.1186/s13049-023-01068-y

**Published:** 2023-03-09

**Authors:** Mohamed Jalloh, Mahdi Sharif-Alhoseini

**Affiliations:** Sina Trauma and Surgery Research Center, Tehran University of Medical Sciences, Tehran, Iran

**Dear Editor,**

With interest, we read the article by Khaki et al. [1]. To identify the most suitable predictive computed tomographic (CT) scoring system for traumatic brain injuries (TBI) patients, they reported that the Stockholm [2] and the Helsinki [3] systems yielded the closest relationship with the actual outcomes.

To our best knowledge, a typical epidural hematoma (EDP) prognosis is good if it is discovered quickly and managed. Therefore the presence of EDH is considered a positive prognostic sign in the Rotterdam [4], Stockholm [2], and the Helsinki [3] CT scoring systems.

Khaki et al. stated that “in the Rotterdam scoring system, the presence of EDH was considered a negative sign and increased the risk of poor outcome” [1], surprisingly. While we know that the absence of EDH is a negative prognostic indicator in the Rotterdam scoring system [4].

It is recommended to revise the analysis and reinterpret the results to ensure the accuracy of the study.

## Data Availability

Data sharing not applicable to this article as no datasets were generated or analyzed during the current study.
