# Supplementary material for: Epidural hematoma, a positive or negative prognostic factor? Letter to the Editor in response to Khaki et al
Source: Scand J Trauma Resusc Emerg Med. 2023 Mar 9;31:12. doi: 10.1186/s13049-023-01068-y (PMC9996836; doi:10.1186/s13049-023-01068-y)
Supplement: Related texts

# Reply: Authors’ response

**Authors:** Djino Khaki, Johan Ljungqvist

**Affiliations:** 2 grid.1649.a 000000009445082X Department of General Surgery, Sahlgrenska University Hospital, Gothenburg, Sweden; 3 grid.1649.a 000000009445082X Department of Neurosurgery, Sahlgrenska University Hospital, Gothenburg, Sweden; 4 grid.8761.8 0000 0000 9919 9582 Department of Clinical Neuroscience, Institute of Neuroscience and Physiology, The Sahlgrenska Academy, University of Gothenburg, Gothenburg, Sweden

**Dear Editor,**

We kindly thank Dr Jalloh and Dr Sharif-Alhoseini for their observations on our article “Selection of CT variables and prognostic models for outcome prediction in patients with traumatic brain injury” published in Scandinavian Journal of Trauma, Resuscitation and Emergency Medicine in July 2021.

The Rotterdam scoring system is made up of different variables that each one, depending on the result, can add a point to the final score which ranges between 1 and 6, where 1 is the lowest risk and 6 is the highest risk of mortality in 6 months post traumatic brain injuries (TBI). In the Rotterdam scoring system, the presence of EDH yields no points whereas the absence of EDH yields one point, thus increasing the risk of mortality when absent.

In the article, we stated that the presence of EDH in the Rotterdam scoring system was considered a negative sign, and thus increased the risk of poor outcome in patients with TBI.

Our interpretation was incorrect since the presence of EDH is not considered an increased risk of mortality. However, the Rotterdam scoring system does not show a decrease in mortality when EDH is present such as in Stockholm and Helsinki CT scoring systems, meaning that there is no impact on outcome, but instead, inversely yields a worse outcome when EDH is absent. Whether one still can interpret EDH as a positive prognostic factor in the Rotterdam CT scoring system is a matter of discussion, because its presence does not make a difference to the risk of mortality.

Our error is clear, we cannot state that EDH is a negative prognostic sign in the Rotterdam CT scoring system. However, the analyses have been reviewed and the calculations were performed correctly; hence, the results were not affected by this error.

We hereby ask the editor to correct the manuscript accordingly.
